# Tophaceous Gout simulating infected Ankle Implants

**DOI:** 10.5704/MOJ.1611.015

**Published:** 2016-11

**Authors:** K Ioannis, P Ippokratis, T Nazzar

**Affiliations:** Department of Orthopaedics, University of Leeds, Leeds, United Kingdom

**Keywords:** Gout, tophi, ankle fracture, implants

## Abstract

Gout is a well known metabolic disorder characterized by the formation of urate crystals in joints resulting in recurrent attacks of acute inflammatory arthritis following which tophi can occur in joints or subcutaneous tissues. We report a rare localization of gouty tophi in a 52 years old male. The tophi had formed over the stainless steel implant used for the fixation of a lateral malleolus fracture 20 years ago.

## Introduction

Gout is a disorder of purine metabolism resulting in the deposition of monosodium urate crystals. It is the most common microcrystalline arthropathy, which affects approximately 5% of the arthritic population, with a worldwide distribution^1^. A chronic form of gout is seen in approximately 10% of patients and is characterized by the formation of nodular masses of monosodium urate crystals (tophi). Tophi can be deposited in different areas of the body with the common locations being the olecranon bursa and first metatarsophalangeal joint. In the presence of urate crystals, a localized and systemic inflammatory state ensues, such as in an acute infection, cytokines and chemokines trigger inflammation and cause arthritis^2^.

## Case Report

**Fig. 1:**
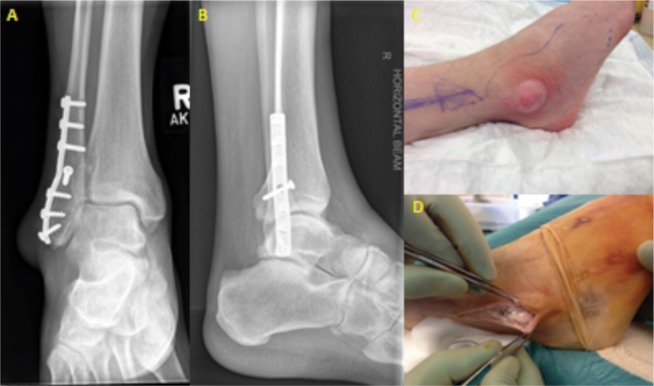
(A) Tophaceous gout simulating infected ankle metalwork. (B) AP and lateral radiograph of the patient’s ankle revealing intact metalwork and no signs of osteomyelitis. (C) Initial presentation characterized by a fluctuant swelling over the lateral malleolus associated with cellulitis. (D) Intraoperative the existence of tophi was noted but no evidence of infection.

A 52 years old male presented to the accident and emergency department of our institution with complaints a painful swelling over the right lateral malleolus for two days. (Fig. 1) This swelling was localized to the distal end of a surgical scar from an open reduction and internal fixation of a Weber B ankle fracture 20 years ago.

Clinical examination revealed a 3x3 cm fluctuant swelling surrounded by soft tissue oedema and erythema over the lateral malleolus. The swelling was inflamed and tender. The erythema was limited to the lateral malleolus and did not extend proximally. The skin was intact. There were no palpable lymph nodes proximally. The ankle movements were significantly restricted due to the pain over the fibular end but the patient was able to fully weight-bear with some discomfort.

The patient was apyrexial and systemically well. His white cell count was normal. His CRP and urate were increased at 50 mg/L (normal <5) and 493 µmol/L (normal 200-430 µmol/L) respectively. Radiographs revealed no recent bony injury with intact screw and plate fixation over the distal fibula. Although there was significant soft tissue swelling over the lateral malleolus, there were no signs of osteomyelitis.

The patient was suffering from osteoarthritis of the ankle requiring non-steroidal anti-inflammatories as necessary and allopurinol 300mg once daily for hyperuricaemia. The patient had sustained a Weber B right ankle fracture 20 years ago, which was treated with a lag screw and a neutralization one-third tubular plate. Since the fixation the patient had experienced occasional mild discomfort but not requiring medical attention.

Based on the clinical findings, an infection involving the implants was suspected and the patient was scheduled for incision and drainage of the removal of the implants.

Intra-operatively, no pus collection was found, no signs of loosening of the implants and no infected bone. The deposit was evacuated, the soft tissues debrided and the implants removed. Samples of the material submitted for microbiology and histology were clear of microorganism but were positive for polymorphs and monosodium urate crystals.

Following surgery, the wound healed well within two weeks. The patient was discharged and no complications were noted at 3 months post-operative follow-up.

## Discussion

Gout represents the commonest cause of crystal-induced inflammatory arthritis in adults. Increased alcohol consumption, modern life style dietary habits, obesity and increased use of drugs causing hyperuricemia are all contributing factors. Monosodium urate crystals can result in localized inflammatory response mediated by the release of cytokines such as TNF, IL-6 and chemokines ligands CxC8 and CxC1^1, 3^. Not infrequently, clinicians have difficulty differentiating between an acute gout attack and septic arthritis as both pathologies share similar symptomatology. To make matters worse, coexistence of gouty and septic arthritis has been reported but it is rare^4^.

In the present case, it is clear that a potential infection process over the surgical implants could have had disastrous complications for the patient. Only during surgery were we able to demonstrate the presence of the tophaceous gout collection over the implants and the absence of the purulent infection with no bony involvement. It was the microbiology report which finally confirmed the presence of monosodium urate crystals, polymorphs and the absence of microorganisms in the cultures. Tophaceous gout involving the tip of the screw head is a rare localization and can simulate an abscess. In the literature, only one previous similar case has been reported in 1990 of tophaceous gout material over implanted metal device after fixation, simulating deep tissue infection^5^.

## Conflict of Interest

The authors report no conflicts of interest and are solely responsible for the content and writing of the clinical report.
